# Supplementary material for: Antibiotic consumption and utilization at a large tertiary care level hospital in Uganda: A point prevalence survey
Source: PLoS One. 2025 Jan 10;20(1):e0313587. doi: 10.1371/journal.pone.0313587 (PMC11723590; doi:10.1371/journal.pone.0313587)
Supplement: Attachment — Submitted filename: PONE-D-24-28507 Review doc.pdf [file pone.0313587.s005.pdf]

PLOS ONE

Antibiotic consumption and utilization at a large tertiary care level hospital in Uganda: a point prevalence survey.

--Manuscript Draft--

**Manuscript Number:**	PONE-D-24-28507
**Article Type:**	Research Article
**Full Title:**	Antibiotic consumption and utilization at a large tertiary care level hospital in Uganda: a point prevalence survey.
**Short Title:**	Antibiotic consumption and utilization study at a large tertiary care hospital in Uganda
**Corresponding Author:**	Mark Kizito<br>SUN: Soroti University<br>Soroti, UGANDA
**Keywords:**	Antibiotics; Defined Daily Dose per 1000 inhabitants per day (DID); Uganda
**Abstract:**	Introduction: Effective antimicrobial stewardship programs require data on antibiotic consumption (AMC) and utilization (AMU) to guide interventions. However, such data is often scarce in low-resource settings. We describe the consumption and utilization of antibiotics at a large tertiary-level hospital in Uganda.<br>Methods: In this cross-sectional study at Kiruddu National Referral Hospital, we analyzed medicine delivery records for the period July 2021 to June 2022, accessed on 12/08/2022, to extract AMC data expressed as defined daily doses (DDDs) per 1000 inhabitants per day (DID). We used the WHO point prevalence survey (PPS) to analyze AMU data with a systematic sampling of OPD patients for a period between June and August 2022, and single-day consecutive sampling for all IPD patients admitted before 8:00 AM on 27/11/2022. AMU data was analyzed as the proportion of individual antibiotic prescriptions, indications for prescriptions, and compliance with the national treatment guidelines. Both AMC and AMU data were categorized by the WHO AWaRe (access, watch, and reserve) criteria.<br>Results: In the year 2021-2022, a total of 6.05 DID of antibiotics were consumed comprising 3.61 DID (59.6%) access, 2.44 DID (40.3%) watch, and 0.003 (0.1%) reserve antibiotics. The most consumed antibiotics comprised penicillin (1.61 DID, 26.7%), cephalosporins (1.51, 25%), and imidazole (1.10 DID, 18.1%).<br>A total of 119/211 (56%) patients in the OPD and 99/172 (57.5%) patients in the IPD were prescribed antibiotics. Of the 158 OPD antibiotic prescriptions, 73 (46.2%) were access, 72 (45.6%) were watch, 0 (0%) were reserve, and 13 (8.2%) were unclassified antibiotics. Of the 162 IPD antibiotic prescriptions, 62 (38.3%) were access, 88 (54.3%) were watch, 01 (0.6%) was reserve and 11 (6.8%) were unclassified antibiotics.<br>Indications for antibiotic prescriptions in the OPD comprised respiratory tract infections (53, 38.1%), urinary tract infections (34, 26.6%), gastrointestinal infections (20, 14.4%), and sepsis (17, 12.2%). The indications for antibiotic prescriptions in the IPD comprised sepsis (28.2%), respiratory tract infections (18.3%), burn wounds (14.1%), and gastrointestinal infections (14.1%).<br>Conclusion: Prescription of watch antibiotics in both OPD and IPD hospital settings was high. Establishment of robust antimicrobial stewardship measures could help improve the rational prescription of antibiotics.
**Order of Authors:**	Mark Kizito
	Darius Owachi
	Falisy Lule
	Laura Jung
	vivian Bazanye
	Ibrahim Mugerwa
	Susan Nabadda
	Charles Kabugo

Powered by Editorial Manager® and ProduXion Manager® from Aries Systems Corporation

<table>
<tr><th colspan="2">Additional Information:</th></tr>
<tr><th>Question</th><th>Response</th></tr>
<tr><td>Financial Disclosure

Enter a financial disclosure statement that describes the sources of funding for the work included in this submission. Review the submission guidelines for detailed requirements. View published research articles from PLOS ONE for specific examples.

This statement is required for submission and will appear in the published article if the submission is accepted. Please make sure it is accurate.

Funded studies
Enter a statement with the following details:
• Initials of the authors who received each award
• Grant numbers awarded to each author
• The full name of each funder
• URL of each funder website
• Did the sponsors or funders play any role in the study design, data collection and analysis, decision to publish, or preparation of the manuscript?

Did you receive funding for this work?</td><td>Yes</td></tr>
<tr><td>Please add funding details.
as follow-up to "Financial Disclosure

Enter a financial disclosure statement that describes the sources of funding for the work included in this submission. Review the submission guidelines for detailed requirements. View published research articles from PLOS ONE for specific examples.

This statement is required for submission and will appear in the published article if the submission is accepted. Please make sure it is accurate.</td><td>This study was funded through collaborations with the SPIDAAR program [funded by the Pfizer/Wellcome Trust foundation and the ESKAPE project [funded through the GIZ GmbH].</td></tr>
</table>

Powered by Editorial Manager® and ProduXion Manager® from Aries Systems Corporation

**Funded studies**<br>Enter a statement with the following details:<br>• Initials of the authors who received each award<br>• Grant numbers awarded to each author<br>• The full name of each funder<br>• URL of each funder website<br>• Did the sponsors or funders play any role in the study design, data collection and analysis, decision to publish, or preparation of the manuscript?<br><br>Did you receive funding for this work?"	
Please select the country of your main research funder (please select carefully as in some cases this is used in fee calculation).<br>as follow-up to "**Financial Disclosure**<br><br>Enter a financial disclosure statement that describes the sources of funding for the work included in this submission. Review the submission guidelines for detailed requirements. View published research articles from *PLOS ONE* for specific examples.<br><br>This statement is required for submission and **will appear in the published article** if the submission is accepted. Please make sure it is accurate.<br><br>**Funded studies**<br>Enter a statement with the following details:<br>• Initials of the authors who received each award<br>• Grant numbers awarded to each author<br>• The full name of each funder<br>• URL of each funder website<br>• Did the sponsors or funders play any role in the study design, data collection and analysis, decision to publish, or preparation of the manuscript?<br><br>Did you receive funding for this work?"	SOUTH AFRICA - ZA
**Competing Interests**	No authors have competing interests

Use the instructions below to enter a competing interest statement for this submission. On behalf of all authors, disclose any competing interests that could be perceived to bias this work—acknowledging all financial support and any other relevant financial or non-financial competing interests.<br><br>This statement is required for submission and **will appear in the published article** if the submission is accepted. Please make sure it is accurate and that any funding sources listed in your Funding Information later in the submission form are also declared in your Financial Disclosure statement.<br><br>View published research articles from *PLOS ONE* for specific examples.<br><br>**NO authors have competing interests**<br><br>Enter: *The authors have declared that no competing interests exist.*<br><br>**Authors with competing interests**<br><br>Enter competing interest details beginning with this statement:<br><br>*I have read the journal's policy and the authors of this manuscript have the following competing interests: [insert competing interests here]*<br><br>* typeset	
**Ethics Statement**<br><br>Enter an ethics statement for this submission. This statement is required if the study involved:<br><br>• Human participants<br>• Human specimens or tissue<br>• Vertebrate animals or cephalopods<br>• Vertebrate embryos or tissues	Ethical approval for the study was obtained from the Uganda National Health Laboratory Services Research and Ethics Committee under reference SN_UNHL-2020-9 and registered with the Uganda National Council for Science and Technology, under reference HS1268ES. Permission to review patient records was sought from the hospital administration of Kiruddu National Referral Hospital

• Field research<br><br>Write "N/A" if the submission does not require an ethics statement.<br><br>General guidance is provided below. Consult the submission guidelines for detailed instructions. **Make sure that all information entered here is included in the Methods section of the manuscript.**	

Powered by Editorial Manager® and ProduXion Manager® from Aries Systems Corporation

**Format for specific study types**<br><br>**Human Subject Research (involving human participants and/or tissue)**<br>• Give the name of the institutional review board or ethics committee that approved the study<br>• Include the approval number and/or a statement indicating approval of this research<br>• Indicate the form of consent obtained (written/oral) or the reason that consent was not obtained (e.g. the data were analyzed anonymously)<br><br>**Animal Research (involving vertebrate animals, embryos or tissues)**<br>• Provide the name of the Institutional Animal Care and Use Committee (IACUC) or other relevant ethics board that reviewed the study protocol, and indicate whether they approved this research or granted a formal waiver of ethical approval<br>• Include an approval number if one was obtained<br>• If the study involved *non-human primates*, add *additional details* about animal welfare and steps taken to ameliorate suffering<br>• If anesthesia, euthanasia, or any kind of animal sacrifice is part of the study, include briefly which substances and/or methods were applied<br><br>**Field Research**<br><br>Include the following details if this study involves the collection of plant, animal, or other materials from a natural setting:<br>• Field permit number<br>• Name of the institution or relevant body that granted permission	
**Data Availability**<br><br>Authors are required to make all data underlying the findings described fully available, without restriction, and from the time of publication. PLOS allows rare exceptions to address legal and ethical concerns. See the PLOS Data Policy and FAQ for detailed information.	Yes - all data are fully available without restriction

A Data Availability Statement describing where the data can be found is required at submission. Your answers to this question constitute the Data Availability Statement and **will be published in the article**, if accepted.<br><br>**Important:** Stating 'data available on request from the author' is not sufficient. If your data are only available upon request, select 'No' for the first question and explain your exceptional situation in the text box.<br><br>Do the authors confirm that all data underlying the findings described in their manuscript are fully available without restriction?	
**Describe where the data may be found in full sentences. If you are copying our sample text, replace any instances of XXX with the appropriate details.**<br><br>• If the data are **held or will be held in a public repository**, include URLs, accession numbers or DOIs. If this information will only be available after acceptance, indicate this by ticking the box below. For example: *All XXX files are available from the XXX database (accession number(s) XXX, XXX.)*.<br>• If the data are all contained **within the manuscript and/or Supporting Information files**, enter the following: *All relevant data are within the manuscript and its Supporting Information files.*<br>• If neither of these applies but you are able to provide **details of access elsewhere**, with or without limitations, please do so. For example:<br><br>*Data cannot be shared publicly because of [XXX]. Data are available from the XXX Institutional Data Access / Ethics Committee (contact via XXX) for researchers who meet the criteria for access to confidential data.*<br><br>*The data underlying the results presented in the study are available from (include the name of the third party*	All relevant data are within the manuscript and its Supporting Information files.

and contact information or URL).<br>• This text is appropriate if the data are owned by a third party and authors do not have permission to share the data.<br><br>* typeset	
Additional data availability information:	

Manuscript Click here to access/download;Manuscript;Manuscript.docx

Antibiotic consumption and utilization at a large tertiary care level hospital in Uganda: a point prevalence survey.

Mark Kizito*, [1, 2,] Darius Owachi[2], Falisy Lule[2], Laura Jung[3], Vivian Bazanye[4], Ibrahim Mugerwa[5], Susan Nabadda[5], Charles Kabugo[2]

Affiliations: [1]Department of Internal Medicine, School of Health Sciences, Soroti University, Soroti, Uganda; [2]Kiruddu National Referral Hospital, Kampala, Uganda; [3]Leipzig University Medical Center, Division of Infectious Diseases and Tropical Medicine, Liebigstraße 20, 04103 Leipzig, Germany; [4]Infectious Diseases Institute, Makerere University, Kampala, Uganda; [5]Department of National Health Laboratories and Diagnostic Services, Ministry of Health, Uganda

Corresponding Author*: mkizito2@gmail.com (MK)

Emails

Mark Kizito: mkizito2@gmail.com/ mkizito@sun.ac.ug

Darius Owachi: darius.jason@gmail.com

Falisy Lule: lufassy@gmail.com

Laura Jung: Laura.Jung@medizin.uni-leipzig.de

Vivian Bazanye: vtwemanye@idi.co.ug

Ibrahim Mugerwa: mugerwaibrahim@gmail.com

Susan Nabadda: sndidde@gmail.com

Charles Kabugo: kabugocharles@hotmail.com

Abstract

Introduction: Effective antimicrobial stewardship programs require data on antibiotic consumption (AMC) and utilization (AMU) to guide interventions. However, such data is often scarce in low-resource settings. We describe the consumption and utilization of antibiotics at a large tertiary-level hospital in Uganda.

Methods: In this cross-sectional study at Kiruddu National Referral Hospital, we analyzed medicine delivery records for the period July 2021 to June 2022, accessed on 12/08/2022, to extract AMC data expressed as defined daily doses (DDDs) per 1000 inhabitants per day (DID). We used the WHO point prevalence survey (PPS) to analyze AMU data with a systematic sampling of OPD patients for a period between June and August 2022, and single-day consecutive sampling for all IPD patients admitted before 8:00 AM on 27/11/2022. AMU data was analyzed as the proportion of individual antibiotic prescriptions, indications for prescriptions, and compliance with the national treatment guidelines. Both AMC and AMU data were categorized by the WHO AWaRe (access, watch, and reserve) criteria.

Results: In the year 2021-2022, a total of 6.05 DID of antibiotics were consumed comprising 3.61 DID (59.6%) access, 2.44 DID (40.3%) watch, and 0.003 (0.1%) reserve antibiotics. The most consumed antibiotics comprised penicillin (1.61 DID, 26.7%), cephalosporins (1.51, 25%), and imidazole (1.10 DID, 18.1%).

A total of 119/211 (56%) patients in the OPD and 99/172 (57.5%) patients in the IPD were prescribed antibiotics. Of the 158 OPD antibiotic prescriptions, 73 (46.2%) were access, 72 (45.6%) were watch, 0 (0%) were reserve, and 13 (8.2%) were unclassified antibiotics. Of the 162 IPD antibiotic prescriptions, 62 (38.3%) were access, 88 (54.3%) were watch, 01 (0.6%) was reserve and 11 (6.8%) were unclassified antibiotics. Indications for antibiotic prescriptions in the OPD comprised respiratory tract infections (53, 38.1%), urinary tract infections (34,

26.6%), gastrointestinal infections (20, 14.4%), and sepsis (17, 12.2%). The indications for antibiotic prescriptions in the IPD comprised sepsis (28.2%), respiratory tract infections (18.3%), burn wounds (14.1%), and gastrointestinal infections (14.1%).

Conclusion: Prescription of watch antibiotics in both OPD and IPD hospital settings was high. Establishment of robust antimicrobial stewardship measures could help improve the rational prescription of antibiotics**.**

INTRODUCTION

Antimicrobial Resistance (AMR) is a growing silent pandemic that threatens all spheres of human health, including biomedicine, behavioral science, and environmental protection (1). The global burden of bacterial antibiotic resistance in 2019 was estimated at 1.27 million attributable deaths and 4.95 million associated deaths, with the greatest proportion affecting the Sub-Saharan African region (2). The negative effects of AMR such as increased healthcare costs, increased length of hospital stays, and increased mortality rates (2–5) are estimated to cause a loss of over $100 trillion in the global economy between 2000 and 2050, disproportionately affecting low- and middle-income countries (4,6,7).

Numerous factors contribute to the development and escalation of AMR including antibiotic misuse or -use, self-medication practices, antibiotic over-prescription, weak AMR diagnostic stewardship systems, non-availability of the right antibiotic formularies for optimal access and incomplete antibiotic treatment by patients (8–10). Addressing these factors has been shown to reduce the spread of AMR (11). The June 2021 G7 meeting's health agenda emphasized the importance of optimizing the use of antibiotics as a tool to combat AMR and improve infection management (1).

In low and middle-income countries, the overuse of antibiotics is a concerning issue, with high rates of antibiotic prescription reported across several countries yet sparsity of data on

consumption and utilization persists (12–15). In Uganda, bacterial infections, such as pneumonia, TB, and sepsis, accounted for 18.4% of hospital-based mortality in the fiscal year 2014/2015 (16), and high rates of antibiotic resistance have been reported (17–19). Recent studies showed high rates of antibiotic resistance among patients hospitalized with post-traumatic or burn wound infections in two large tertiary hospitals in Uganda (20,21).

It is therefore imperative to develop effective strategies to curb this growing burden of AMR that poses significant public health and socio-economic importance. Antimicrobial stewardship measures such as the use of antibiograms have been shown to effectively improve antibiotic prescribing in acute-care settings thus reducing the spread of AMR (22). However, to inform effective policies and interventions that optimize use and promote equitable access to medicines, it is essential to document trends on antibiotic consumption and utilization in all settings (23). This study therefore aimed at determining the prevalence of antibiotic consumption and utilization at a large public tertiary care facility in Uganda.

METHODS

Study setting and design.

We conducted a cross-sectional study at Kiruddu National Referral Hospital (KNRH), a 200-bed capacity public healthcare facility located in the southern suburb region of the capital Kampala, Uganda for the period 2021-2022. The hospital is a tertiary-level health facility that provides both medical and surgical in-patient and outpatient department services, diagnostic services, and the largest renal replacement therapy unit in the country. Approximately 100,000 patient visits are received at KNRH each year (24).

In 2021, KNRH established an Antimicrobial Resistance (AMR) Surveillance and Capacity Building program through collaborations with the SPIDAAR program funded by the

Pfizer/Wellcome Trust foundation (25), and the ESKAPE project funded through the German Alliance on Global Health Research (GLOHRA) (26,27). One key output was to determine the baseline antibiotic consumption and utilization patterns of the hospital, as a basis for monitoring the impact of antimicrobial stewardship programs.

Data collection

Antibiotic Consumption

All data was accessed on the 12th of August 2022 and was fully anonymized before patient records were accessed.

KNRH receives a cyclic supply of medicines through the National Medical Stores (NMS), the official government entity that procures and supplies medicines to all public health facilities in Uganda (28). We reviewed invoices and delivery logs for medicines supplied to KNRH by NMS for the fiscal year running from July 2021 through June 2022. Data on quantities and types of antibiotics delivered and their respective prices were extracted and analyzed.

Antibiotic consumption data was presented as the defined daily dose (DDD) which was calculated by dividing the total grams of the antibiotic consumed by the standard (29). The DDDs per 1000 inhabitants per day (DID) were calculated by multiplying the DDDs for the antibiotic by 1000 and dividing the product by the Ugandan population for the year 2022 estimated at 44,200,000 (30). The antibiotics were subcategorized by individual antibiotics, by the WHO AWaRe system, and by the total expenditure of individual and all antibiotics purchased in the fiscal year 2021-2022 (31).

Antibiotic Utilization

We reviewed patient records and registers from the outpatient (OPD) and inpatient (IPD) departments of the hospital to extract data on antibiotic utilization. Antibiotic utilization was

analyzed as the proportion of patients in whom an antibiotic was prescribed, the type of antibiotics prescribed, the indication (diagnosis) for the antibiotic prescriptions, the proportion of missed doses, and the compliance with the national treatment guidelines (32).

For the outpatient data, a sample size of 200 patient records was determined as guided by the WHO PPS methodology. We recruited every 44th patient who visited the OPD between 1st June through 31st August 2022 by systematic sampling until the sample size was achieved. For inpatient data, given the small number of hospital beds at 200, we reviewed all records of patients admitted at the hospital before 8:00 AM of the day of the survey, as guided by the WHO point prevalence survey guidelines, to obtain the recommended sample size (23). The IPD survey was conducted on the 27th of November 2022.

Data on antibiotic prescriptions, indications, dosages, routes of administration, individual wards, missed doses, and medical history were extracted using an open data kit (ODK) tool designed based on the WHO point prevalence survey methodology.

Infectious diagnoses were classified as: a) respiratory tract infections (to include sinusitis, bronchitis, pharyngitis, and/or pneumonia); b) gastrointestinal tract infections (to include gastritis, enterocolitis, appendicitis); c) urinary tract infections (to include uncomplicated or complicated urinary tract infections, pyelonephritis, cystitis, and urethritis), d) central nervous system infections (to include meningitis, encephalitis); skin and soft tissue infections (to include cellulitis, skin abscesses, burns wounds etc.); malaria (where microbiologic confirmation of malaria was made); tuberculosis (where microbiologic confirmation of TB bacilli was made or patient was taking anti-TB medication); and sepsis (bloodstream infection with evidence of organ dysfunction).

Data analysis and management

We coded and double-entered data into Microsoft Excel to remove any inconsistencies and analyzed using descriptive statistics such as frequencies.

Ethical Consideration

Ethical approval for the study was obtained from the Uganda National Health Laboratory Services Research and Ethics Committee under reference SN_UNHL-2020-9 and registered with the Uganda National Council for Science and Technology, under reference **HS1268ES.** Permission to review patient records was sought from the hospital administration of KNRH. The requirement for Informed consent was waived by the IRB and all data was fully anonymized before patient records were accessed.

RESULTS

Characteristics of study patients

A total of 211 patients in the OPD and 172 patients in the IPD were enrolled in the study. Females comprised 130 (61.6%) in the OPD and 100 (58.1%) in the IPD. The median (IQR) age was 32 (15-48) years in the OPD and 40 (26-60.5) years in the IPD.

Infectious diseases were the most common diagnoses encountered in both outpatient (150/183, 82%) and in-patient departments (67/69, 97%). Among the comorbidities, hypertension (23, 48.9%), diabetes mellitus (7, 14.9%), and liver disease (5, 10.6%) were the most common among outpatients while HIV (21, 44.7%), liver disease (7, 14.7%), and chronic kidney disease (5, 10.6%) were prevalent among admitted patients (**Table 1**)

Table 1: Baseline Characteristics of Patients

Variable	OPD (%)	IPD (%)
Total Patients	211	172
Sex, Female	130 (61.6)	100 (58.1)
Age, Median (Interquartile range)	32 (15-48)	40 (26-60.5)

**Antibiotic Therapy**		
Patients on Antibiotics	119 (56.4)	99 (57.6)
Total number of Antibiotics Prescribed	158	162
Antibiotics per encounter	1.33	1.64
**Diagnoses**		
Burn Wounds	0 (0)	10 (14.5)
Central nervous system/Meningitis infections	0 (0)	3 (4.3)
Gastrointestinal Infection	20 (10.9)	7 (10.1)
Malaria	13 (7.1)	2 (2.9)
Peptic ulcer disease	17 (9.3)	2 (2.9)
Peripheral neuropathy	16 (8.7)	0 (0)
Pulmonary tuberculosis	2 (1.1)	3 (4.3)
Respiratory tract infection	53 (29.0)	13 (18.8)
Sepsis	17 (9.3)	14 (20.3)
Skin infections	11 (6.0)	9 (13.0)
Urinary Tract Infection	34 (18.6)	6 (8.7)
Total	183 (100)	69 (100)
**Comorbidities**		
Asthma	4 (8.5)	2 (4.3)
Chronic kidney disease	1 (2.1)	5 (10.6)
Chronic obstructive pulmonary disease	3 (6.4)	3 (6.4)
Cardiovascular disease (Stroke, heart disease)	3 (6.4)	3 (6.4)
Diabetes Mellitus	7 (14.9)	3 (6.4)
HIV	1 (2.1)	21 (44.7)
Hypertension	23 (48.9)	3 (6.4)
Liver disease	5 (10.6)	7 (14.9)
Total	47 (100)	47 (100)

OPD – outpatient department, IPD – inpatient department

Antibiotic consumption and expenditure

A total of 267,595 DDDs of antibiotics were consumed in the year 2021/2022 at KNRH with a total DID of 6.05 DDDs per 1,000 inhabitants per day. By the AWaRe classification, 3.61 (59.6%) DID were access, 2.44 (40.3%) DID were watch and 0.003 (0.1%) DID were reserve antibiotics. By antibiotic classes, the most consumed antibiotics comprised penicillin 1.61 DID (26.7%), cephalosporins 1.51 DID (25%), and imidazole 1.10 (18.1%); all collectively

comprising more than two-thirds of the total antibiotics consumed in the year 2021-2022. Of the individual antibiotics, the most consumed comprised metronidazole (1.19 DID, 19.6%), ceftriaxone (0.83 DID, 13.6%), amoxicillin-clavulanate (0.70 DID, 11.5%), cefixime (0.68 DID, 11.2%), and amoxicillin (0.68 DID, 11.2%), collectively contributing to 4.08 DID (67.2%) of total antibiotics consumed (**Table 2**).

In the fiscal year 2021-2022, total expenditure on antibiotics was $110,785, which comprised 16% of the total hospital budget expenditure of $682,985. The bulk of antibiotic expenditure was on watch antibiotics costing $66,527 (60%) and access antibiotics costing $35,029 (31.6%) while the least expenditure was on reserve antibiotics at $3,939 (3.6%) and unclassified antibiotics at $5,290 (4.8%). By individual drugs, the most expenditure was on ceftriaxone $31,094 (28.1%), metronidazole $11,483 (10.4%), levofloxacin $10,880 (9.8%), and amoxicillin-clavulanic acid $9,722 (8.8%) **(Table 2)**.

Table 2: Antibiotic consumption and expenditure during the fiscal year 2021/2022.

Antibiotic Consumption	DDD (%)	DID	Expenditure* (%)
**AWaRe classification**			
Access	159,599 (59.6)	3.61	35,029 (31.6)
Watch	107,846 (40.3)	2.44	66,527 (60.1)
Reserve	150 (0.1)	0.00	3,939 (3.6)
Unclassified	0 (0)	0	5,290 (4.8)
Total	267,595 (100)	6.05	110,785 (100)
**Antibiotic class**		0	
Penicillin	71,308 (26.6)	1.61	25,797 (23.3)
Cephalosporins	66,800 (25.0)	1.51	38,263 (34.5)
Imidazole	48,501 (18.1)	1.10	11,483 (10.4)
Tetracyclines	25,000 (9.3)	0.57	473 (0.4)
Fluoroquinolones	23,885 (8.9)	0.54	12,933 (11.7)
Macrolides	19,840 (7.4)	0.45	5,800 (5.2)
Sulfonamides	10,890 (4.1)	0.25	0 (0)
Carbapenems	625 (0.2)	0.01	11,806 (10.7)
Aminoglycosides	600 (0.2)	0.01	2,565 (2.3)
Glycopeptides	146 (0.1)	0.00	1,664 (1.5)
Total	267,595 (100)	6.05	110,785 (100)
**Individual Antibiotics**		0	
Amikacin	600 (0.2)	0.01	2,565 (2.3)
Amoxicillin	30,000 (11.2)	0.68	1,984 (1.8)
Amoxicillin-Clavulanate	30,858 (11.5)	0.70	9,722 (8.8)
Ampicillin-Cloxacillin	8,062 (3.0)	0.18	1,591 (1.4)

Azithromycin	12,500 (4.7)	0.28	1,040 (0.9)
Cefixime	30,000 (11.2)	0.68	6,219 (5.6)
Cefotaxime	301 (0.1)	0.01	950 (0.9)
Ceftriaxone	36,500 (13.6)	0.83	31,094 (28.1)
Ciprofloxacin	13,020 (4.9)	0.29	2,053 (1.9)
Clarithromycin	5,040 (1.9)	0.11	4,412 (4.0)
Cotrimoxazole	10,890 (4.1)	0.25	0 (0)
Doxycycline	25,000 (9.3)	0.57	473 (0.4)
Erythromycin	3,000 (1.1)	0.07	348 (0.3)
Flucloxacillin-Amoxicillin	0 (0)	0	5,290 (4.8)
Imipenem	150 (0.1)	0.00	3,939 (3.6)
Levofloxacin	6,865 (2.6)	0.16	10,880 (9.8)
Meropenem	475 (0.2)	0.01	7,867 (7.1)
Metronidazole	52,501 (19.6)	1.19	11,483 (10.4)
Penicillin-Benzathine	980 (0.4)	0.02	724 (0.7)
Piperacillin-Tazobactam	707 (0.3)	0.02	6,487 (5.9)
Vancomycin	146 (0.1)	0.00	1,664 (1.5)
Total	267,595 (100)	6.05	110,785 (100)

DDD: Defined daily dose, DID: DDD per 1000 inhabitants per day, *Expenditure in US Dollars

Antibiotic Utilization in the Outpatient Department

Of the 211 patients reviewed from the OPD, 119 (56%) were prescribed antibiotics. A total of 158 antibiotics were prescribed, with an average of 1.33 antibiotic prescriptions per encounter (158/119). However, only 35/119 (29%) of the patients received appropriate antibiotic prescriptions in accordance with the national treatment guidelines.

Of the 158 antibiotic prescriptions, 73 (46.2%) comprised access antibiotics while 72 (45.6%) comprised watch antibiotics. No reserve antibiotics were prescribed in the OPD. By antibiotic classes, the most prescribed antibiotics included penicillin (44, 27.8%), cephalosporins (40, 25.3%), and imidazole (29, 18.4%). By individual antibiotics, cefixime (33, 20.9%), metronidazole (28, 17.7%), amoxicillin-clavulanate (14, 8.9%), and azithromycin (13, 8.2%) were the most prescribed antibiotics.

The common indications for antibiotic prescriptions in the OPD included respiratory tract infections (53, 38.1%), urinary tract infections (34, 26.6%), gastrointestinal infections (20, 14.4%), and sepsis (17, 12.2%). (**Table 3**).

Antibiotic utilization in the In-patient Department (IPD)

Of the 172 patients who were reviewed in the IPD, 99 (57.5%) were prescribed antibiotics. A total of 162 antibiotics were prescribed with an average of 1.63 antibiotic prescriptions per encounter (162/99). However, only 31/99 (31%) of the patients received appropriate antibiotic prescriptions in accordance with the national treatment guidelines.

By the AWaRe classification, 62 (38.3%) were access, 88 (54.3%) were watch, 01 (0.6%) were reserve while 11 (6.8%) were unclassified antibiotics. By antibiotic classification, the most prescribed antibiotics in the IPD included cephalosporins (63, 38.9%), imidazole (39, 24.1%), and fluoroquinolones (19, 11.7%). By individual antibiotics, ceftriaxone (60, 37%), metronidazole (39, 24.1%), and levofloxacin (15, 9.3%) were the most prescribed.

The common indications for antibiotic prescriptions in the IPD comprised sepsis (20, 28.2%), respiratory tract infections (13, 18.3%), gastrointestinal infections (10, 14.1%), and burn wound infections (10, 14.1%) (**Table 3**).

Table 3: Antibiotic utilization by AWARE classification, antibiotic class, prescribed antibiotics, and indications for antibiotic use.

Antibiotic Utilization	OPD (%)	IPD (%)
**Utilization by AWaRe Classification**		
Access	73 (46.2)	62 (38.3)
Watch	72 (45.6)	88 (54.3)
Reserve	0 (0)	1 (0.6)
Unclassified	13 (8.2)	11 (6.8)
Total	158 (100)	162 (100)
**Utilization by Antibiotic class**		
Penicillin	44 (27.8)	23 (14.2)
Cephalosporins	40 (25.3)	63 (38.9)
Imidazole	29 (18.4)	39 (24.1)
Macrolides	18 (11.4)	3 (1.9)
Fluoroquinolones	15 (9.5)	19 (11.7)
Tetracyclines	8 (5.1)	1 (0.6)
Sulfonamides	0 (0)	10 (6.2)
Aminoglycosides	0 (0)	2 (1.2)
Glycopeptides	0 (0)	1 (0.6)
Nitrofurantoin	2 (1.3)	1 (0.6)
Amphenicols	2 (1.3)	0 (0)
Total	158 (100)	162 (100)

**Utilization by Individual Antibiotics**		
Amoxicillin	13 (8.2)	1 (0.6)
Amoxicillin-clavulanate	14 (8.9)	2 (1.2)
Azithromycin	13 (8.2)	3 (1.9)
Ampicillin-cloxacillin	11 (7.0)	5 (3.1)
Benzyl penicillin	0 (0)	2 (1.2)
Cefixime	33 (20.9)	1 (0.6)
Cefotaxime	0 (0)	2 (1.2)
Cefpodoxime	1 (0.6)	0 (0)
Ceftriaxone	4 (2.5)	60 (37.0)
Cefuroxime	2 (1.3)	0 (0)
Chloramphenicol	2 (1.3)	0 (0)
Ciprofloxacin	5 (3.2)	4 (2.5)
Clarithromycin	3 (1.9)	0 (0)
Cotrimoxazole	0 (0)	10 (6.2)
Doxycycline	8 (5.1)	1 (0.6)
Erythromycin	2 (1.3)	0 (0)
Flucloxacillin	6 (3.8)	9 (5.6)
Gentamycin	0 (0)	2 (1.2)
Levofloxacin	10 (6.3)	15 (9.3)
Metronidazole	28 (17.7)	39 (24.1)
Nifuroxazide	2 (1.3)	0 (0)
Nitrofurantoin	0 (0)	1 (0.6)
Piperacillin-tazobactam	0 (0)	4 (2.5)
Tinidazole	1 (0.6)	0 (0)
Vancomycin	0 (0)	1 (0.6)
Total	158 (100)	162 (100)
**Indications for antibiotic use**		
Respiratory tract Infection	53 (38.1)	13 (18.3)
Urinary tract Infection	37 (26.6)	6 (8.5)
Sepsis	17 (12.2)	20 (28.2)
Gastrointestinal infection	20 (14.4)	10 (14.1)
Central nervous system infection	0 (0)	3 (4.2)
Burn Wound Infection	0 (0)	10 (14.1)
Medical prophylaxis	12 (8.6)	7 (9.9)
Surgical prophylaxis	0 (0)	2 (2.8)
Total	139 (100)	71 (100)

OPD – outpatient department, IPD – inpatient department

DISCUSSION

In this study, we assessed antibiotic consumption and utilization at a large tertiary hospital in Uganda. We found a relatively high rate of antibiotic prescription with more than half of patients receiving antibiotic prescriptions in both OPD and IPD settings. More than one-quarter of a million DDDs of antibiotics were consumed annually, with three-fifths comprising access

antibiotics and two-fifths comprising watch antibiotics but negligible reserve antibiotics. The total DDDs per 1000 inhabitants per day were 6.05. However, there were more prescriptions of watch antibiotics in the IPD compared to the OPD, and generally low compliance with the national treatment guidelines in both OPD and IPD settings.

Antibiotic consumption

The overall consumption of antibiotics at Kiruddu National Referral Hospital in the financial 2021-2022 of 6.05 DID was significantly lower than what was reported as the national average consumption of 29.02 for all antimicrobials for the year 2021 (33). To the best of our knowledge, this could be one of the first studies to report on antibiotic consumption from a public tertiary care facility in Uganda. However, we believe that this study provides a baseline for measuring trends in antibiotic consumption at KNRH which can be used to evaluate the performance of antimicrobial stewardship programs to reduce antibiotic use.

The most consumed antibiotic classes were penicillin, cephalosporins, and imidazole, which are consistent with other studies done in multiple countries reporting data to WHO GLASS (34). For example, a South African study reported that penicillin and imidazole were the most commonly consumed antibiotic classes from 2014 to 2018 (35). Another study done among 14 Kenyan hospitals also reported similar findings (36). By contrast, the most consumed antibiotic class in Tanzania was tetracycline (37). The differences in the Ugandan and Tanzanian national treatment guidelines as well as different procurement patterns in public and private health sectors could explain this observation (33).

In our study, about 60% of the IPD antibiotic consumption were access antibiotics. Other studies conducted in Uganda (33,38), Ethiopia (39), and Tanzania (37) revealed similar findings. The observed antibiotic consumption for access antibiotics at KNRH also compares

with the WHO AWaRe recommendation of at least 60% access antibiotics in total antibiotic consumption (40).

The WHO's AWaRe classification promotes narrow-spectrum antibiotics over broad-spectrum ones to curb antibiotic resistance (41,42). Our study revealed that Watch category antibiotics exceeded recommended levels of 40% (41),comprising 45.6% and 54.3% of overall antibiotic use in OPD and IPD, respectively. This highlights the urgency for robust antimicrobial stewardship protocols to improve prescription practices. Adherence to AWaRe guidelines can mitigate selection pressure and the spread of resistance, ensuring the effectiveness of antibiotics.

The limited consumption of reserve antibiotics in our study is a predictable outcome due to the public procurement system being centered on primary healthcare (38), as well as the prohibitive cost of reserve antibiotics which makes them unaffordable for a sizable portion of the population. Additionally, Kiruddu National Referral Hospital implemented a policy restricting the dispensing of reserve antibiotics to instances where culture and sensitivity results demonstrate the necessity for such antibiotics. While the overall minimal use of reserve antibiotics is encouraging in terms of antibiotic stewardship, it could also potentially suggest lack of access for patients who may require them in situations where resistance to first- and second-line treatment options emerge (21,43).

Antibiotic Utilization: High Antibiotic Prescriptions

We found that one in every two patients visiting the OPD or admitted to the IPD were prescribed antibiotics. The high antibiotic prescription rate observed in this study is higher than the recommended WHO target of 40% (23). A possible explanation might be due to the high burden of infectious diseases reported in Uganda, resulting in increased risk for hospitalization and mortality (44,45). However, overuse of antibiotics due to lack of awareness and training in

health care staff might also play a role. Our findings also compare with studies done in other public healthcare facilities in Uganda (46,47), as well as other Sub-Saharan African countries (13,14,48) where the burden of infectious diseases is well documented. Unfortunately, the over-prescription of antibiotics may contribute to the propagation of antibiotic resistance (49), with the Sub-Saharan African region projected to suffer the greatest impact of AMR (2).

Data on antibiotic resistance patterns show high rates of resistance to commonly prescribed antibiotics, particularly ceftriaxone (21,50). Several studies reveal increasing patterns of antimicrobial resistance to common antibiotics used in the local settings. Developing and strengthening of antimicrobial stewardship measures such as the use of antibiograms or adopting evidence-based empirical treatment guidelines could guide clinicians on the rational use of antibiotic prescriptions and help reduce the spread of AMR (22,51,52).

Antimicrobial Utilization: indications for antibiotic use

In our study, the commonly prescribed antibiotics in the IPD included ceftriaxone, metronidazole, levofloxacin, and cotrimoxazole while cefixime, metronidazole, amoxicillin-clavulanic acid, and azithromycin were the most prescribed in the OPD. Several studies in Uganda have reported similar patterns of prescription of these antibiotics (12,20,46,47,53), a trend also observed in other countries in the Sub-Saharan region (13,54,55). One possible explanation for this similarity in prescriptions is the listing of these antimicrobial agents on the WHO list of essential medicines (56) that often guide local treatment guidelines (32). Levofloxacin is often reserved for the treatment of drug-resistant infections such as multi-drug resistant tuberculosis (32,57), but studies indicate a tendency to inappropriately prescribe it in non-TB infections (58).

In this study, the indications for antibiotic use were respiratory tract infections, urinary tract infections, sepsis, gastrointestinal infections, and burn wound infections. This agrees with what

has been reported by other studies done in Uganda (12,44,45) and other countries in Sub-Saharan Africa (54,55) that further highlight the burden of infectious diseases in low-resource settings.

Compliance with Treatment Guidelines

The proportion of antibiotic prescriptions that aligned with the recommended national treatment guidelines was low, 29% and 31% for outpatient and inpatient departments, respectively. Worryingly, this pattern is also observed in other regions of the country. One study investigating antibiotic prescription practices in Eastern Uganda reported only 17% of antibiotic prescriptions being compliant in accordance with the Uganda Clinical Guidelines (59). Another point prevalence survey across 13 hospitals in Uganda found 30% compliance with Uganda Clinical Guidelines (60).

The reasons for poor compliance with the national treatment guidelines in our study are unclear. However, common reasons for poor compliance with the national treatment guidelines include poor dissemination of the guidelines, lack of diagnostics such as in microbiology and radiology, long turn-around times for laboratory results, prescribers' preferences and behaviors, and influence of pharmacies and pharmaceutical companies (58,61). The poor compliance with national guidelines could be a contributing factor to the high prescription rate of the Watch category group of antibiotics (2). In contrast, compliance with national guidelines in Tanzania and South Africa was high at 84% and 90.2% respectively (62,63). The relatively high compliance with national guidelines could partly be attributed to the availability of robust national antimicrobial stewardship programs; along with strong healthcare quality improvement initiatives (64,65). These are potentially useful strategies for Uganda to adopt, to improve compliance with treatment guidelines. KNRH currently undergoes efforts to improve AMS and local treatment recommendations have been developed and disseminated amongst the health care staff. These measures, alongside regular training, are aimed at improving AMU.

Strengths and Limitations

The findings of this study provide a basis for identifying gaps to improve antimicrobial stewardship practices at Kiruddu Hospital. The use of the standard WHO point prevalence survey (PPS) methodology allows for comparisons with future studies to monitor trends in antibiotic prescription practices, which could be used to evaluate current AMS measures and influence critical health policy-making decisions, e.g., concerning procurement. It also builds on previous efforts by the Uganda National Drug Authority and provides updated information on Antimicrobial consumption to support policy discussions at the local and national level during action plan reviews on AMR. However, we acknowledge some inherent weaknesses of this study. Firstly, the findings may not be readily generalizable to other healthcare settings since this was a single-site study at a tertiary-level healthcare facility. Secondly, the point-in-time nature of the PPS design further limits insight into seasonal patterns in antibiotic use. Nonetheless, we believe that the findings provide valuable lessons for improving antimicrobial stewardship practices.

Conclusion

Antibiotic use, particularly for watch antibiotics was high, and was associated with poor compliance to national treatment guidelines. Establishment of robust antimicrobial stewardship measures could help improve and monitor the rational prescription of antibiotics.

LIST OF ABBREVIATIONS

AMR-	Antimicrobial Resistance
ASP-	Antibiotic Stewardship Program
AWaRe	Access, Watch, Reserve
CDC-	Centers for Disease Control and Prevention
CKD-	Chronic Kidney Disease
CVD-	Cardiovascular Disease

DDD-	Defined Daily Dose
DID-	Defined Daily Doses per 1000 inhabitants per day
HIV-	Human Immunodeficiency Virus
IV-	Intravenous
GIT-	Gastrointestinal
IPD-	In-patient Department
KNRH-	Kiruddu National Referral Hospital
ODK-	Open Data Kit
OPD-	Outpatient Department
PPS-	Point Prevalence Survey
RTI-	Respiratory tract infection
USA-	United States of America
UCG-	Uganda Clinical Guidelines
UTI-	Urinary tract infection
WHO-	World Health Organization

DECLARATIONS

Ethics approval and consent to participate.

Ethical approval for the study was obtained from the Uganda National Health Laboratory Services Research and Ethics Committee under reference SN_UNHL-2020-9 and registered with the Uganda National Council for Science and Technology, under reference **HS1268ES.** Permission to review patient records was sought from the hospital administration of KNRH. The requirement for Informed consent was waived by the IRB and all data was fully anonymized before patient records were accessed.

Consent for publication

Not applicable

Availability of data and materials

The datasets used and/or analyzed during the current study are available from the corresponding author on reasonable request.

Competing interests

The authors declare that they have no competing interests.

Funding

This study was funded through collaborations with the SPIDAAR program [funded by the Pfizer/Wellcome Trust foundation and the ESKAPE project [funded through the GIZ GmbH].

Authors' contributions

The study concept and design were by MK, FL, DO, LJ, IB, SN, and CK. MK, VB, and FL collected the data. MK, FL, VB, and DO analyzed the data. All authors read and approved the final manuscript.

Acknowledgments

We are indebted to the Kiruddu National Referral Hospital staff and patients who participated in the study.

REFERENCES

1. Charani E, McKee M, Ahmad R, Balasegaram M, Bonaconsa C, Merrett GB, et al. Optimizing antimicrobial use in humans - review of current evidence and an interdisciplinary consensus on key priorities for research. The Lancet regional health Europe [Internet]. 2021 Aug 1 [cited 2023 Sep 9];7. Available from: https://pubmed.ncbi.nlm.nih.gov/34557847/

2. Murray CJ, Ikuta KS, Sharara F, Swetschinski L, Robles Aguilar G, Gray A, et al. Global burden of bacterial antimicrobial resistance in 2019: a systematic analysis. Lancet [Internet]. 2022 Feb 12 [cited 2022 Oct 5];399(10325):629–55. Available from: https://pubmed.ncbi.nlm.nih.gov/35065702/

3. Cassini A, Högberg LD, Plachouras D, Quattrocchi A, Hoxha A, Simonsen GS, et al. Attributable deaths and disability-adjusted life-years caused by infections with antibiotic-resistant bacteria in the EU and the European Economic Area in 2015: a population-level modelling analysis. Lancet Infect Dis [Internet]. 2019 Jan 1 [cited 2023 Sep 9];19(1):56–66. Available from: https://pubmed.ncbi.nlm.nih.gov/30409683/

4. Founou RC, Founou LL, Essack SY. Clinical and economic impact of antibiotic resistance in developing countries: A systematic review and meta-analysis. PLoS One [Internet]. 2017 Dec 1 [cited 2023 Sep 9];12(12). Available from: https://pubmed.ncbi.nlm.nih.gov/29267306/

5. Saleem Z, Godman B, Cook A, Khan MA, Campbell SM, Seaton RA, et al. Ongoing Efforts to Improve Antimicrobial Utilization in Hospitals among African Countries and Implications for the Future. Antibiotics (Basel) [Internet]. 2022 Dec 1 [cited 2023 Sep 9];11(12). Available from: https://pubmed.ncbi.nlm.nih.gov/36551481/

6. Collignon P. Antimicrobial resistance. Emerg Infect Dis [Internet]. 2000 [cited 2023 Sep 9];6(4):434–6. Available from: https://pubmed.ncbi.nlm.nih.gov/10905989/

7. Hofer U. The cost of antimicrobial resistance. Nat Rev Microbiol [Internet]. 2019 Jan 1 [cited 2023 Sep 9];17(1):3. Available from: https://pubmed.ncbi.nlm.nih.gov/30467331/

8. Mugerwa I, Nabadda SN, Midega J, Guma C, Kalyesubula S, Muwonge A. Antimicrobial resistance situational analysis 2019–2020: Design and performance for human health surveillance in Uganda. Trop Med Infect Dis [Internet]. 2021 Dec 1 [cited 2022 Oct 5];6(4). Available from: /pmc/articles/PMC8544686/

9. Browne AJ, Chipeta MG, Haines-Woodhouse G, Kumaran EPA, Hamadani BHK, Zaraa S, et al. Global antibiotic consumption and usage in humans, 2000-18: a spatial modelling study. Lancet Planet Health [Internet]. 2021 Dec 1 [cited 2023 Sep 9];5(12):e893–904. Available from: https://pubmed.ncbi.nlm.nih.gov/34774223/

10. Baraka MA, Alboghdadly A, Alshawwa S, Elnour AA, Alsultan H, Alsalman T, et al. Perspectives of Healthcare Professionals Regarding Factors Associated with Antimicrobial Resistance (AMR) and Their Consequences: A Cross Sectional Study in Eastern Province of Saudi Arabia. Antibiotics (Basel) [Internet]. 2021 Jul 1 [cited 2023 Sep 9];10(7). Available from: https://pubmed.ncbi.nlm.nih.gov/34356799/

11. Holmes AH, Moore LSP, Sundsfjord A, Steinbakk M, Regmi S, Karkey A, et al. Understanding the mechanisms and drivers of antimicrobial resistance. Lancet [Internet]. 2016 Jan 9 [cited 2023 Sep 9];387(10014):176–87. Available from: https://pubmed.ncbi.nlm.nih.gov/26603922/

12. Kizito M, Lalitha R, Kajumbula H, Ssenyonga R, Muyanja D, Byakika-Kibwika P. Antibiotic Prevalence Study and Factors Influencing Prescription of WHO Watch Category Antibiotic Ceftriaxone in a Tertiary Care Private Not for Profit Hospital in

Uganda. Antibiotics (Basel) [Internet]. 2021 Oct 1 [cited 2023 Feb 25];10(10). Available from: https://pubmed.ncbi.nlm.nih.gov/34680748/

13. D'Arcy N, Ashiru-Oredope D, Olaoye O, Afriyie D, Akello Z, Ankrah D, et al. Antibiotic Prescribing Patterns in Ghana, Uganda, Zambia and Tanzania Hospitals: Results from the Global Point Prevalence Survey (G-PPS) on Antimicrobial Use and Stewardship Interventions Implemented. Antibiotics (Basel) [Internet]. 2021 Sep 1 [cited 2023 Sep 9];10(9). Available from: https://pubmed.ncbi.nlm.nih.gov/34572704/

14. Fentie AM, Degefaw Y, Asfaw G, Shewarega W, Woldearegay M, Abebe E, et al. Multicentre point-prevalence survey of antibiotic use and healthcare-associated infections in Ethiopian hospitals. BMJ Open [Internet]. 2022 Feb 11 [cited 2023 Sep 9];12(2). Available from: https://pubmed.ncbi.nlm.nih.gov/35149567/

15. Kalungia AC, Mukosha M, Mwila C, Banda D, Mwale M, Kagulura S, et al. Antibiotic Use and Stewardship Indicators in the First- and Second-Level Hospitals in Zambia: Findings and Implications for the Future. Antibiotics (Basel) [Internet]. 2022 Nov 1 [cited 2023 Sep 9];11(11). Available from: https://pubmed.ncbi.nlm.nih.gov/36421270/

16. World Health Organization; Uganda: Antimicrobial resistance national action plan 2018-2023 [Internet]. 2018 [cited 2022 Oct 9]. Available from: https://www.who.int/publications/m/item/uganda-antimicrobial-resistance-national-action-plan-2018-2023

17. H K, AW F, O M, C N, C I, A A, et al. Antimicrobial Drug Resistance in Blood Culture Isolates at a Tertiary Hospital, Uganda. Emerg Infect Dis [Internet]. 2018 Jan 1 [cited 2021 Sep 14];24(1):174–5. Available from: https://pubmed.ncbi.nlm.nih.gov/29260682/

18. Lubwama M, Phipps W, Najjuka CF, Kajumbula H, Ddungu H, Kambugu JB, et al. Bacteremia in febrile cancer patients in Uganda. BMC Res Notes [Internet]. 2019 Jul 30 [cited 2023 Sep 9];12(1). Available from: https://pubmed.ncbi.nlm.nih.gov/31362783/

19. D H, L A, C O, E M, H T, RO A. Antimicrobial resistance in pathogenic aerobic bacteria causing surgical site infections in Mbarara regional referral hospital, Southwestern Uganda. Sci Rep [Internet]. 2019 Dec 1 [cited 2021 Sep 14];9(1). Available from: https://pubmed.ncbi.nlm.nih.gov/31754237/

20. Jung L, Kiwanuka J, Mbabazi L, Nakate V, Musaazi J, Nabajja H, et al. A case for routine microbial diagnostics: Results from antimicrobial susceptibility testing in post-traumatic wound infections at a Ugandan tertiary care hospital. Bhadricha HB, editor. PLOS global public health [Internet]. 2023 Aug 15 [cited 2023 Sep 9];3(8):e0001880. Available from: https://pubmed.ncbi.nlm.nih.gov/37582103/

21. Weinreich J, Namatovu C, Nsibirwa S, Mbabazi L, Kajumbula H, Dietze N, et al. Microbiological Findings and Clinical Outcomes in Ugandan Patients with Infected Burn Wounds. European Burn Journal 2023, Vol 4, Pages 69-79 [Internet]. 2023 Feb 7 [cited 2023 Feb 25];4(1):69–79. Available from: https://www.mdpi.com/2673-1991/4/1/7/htm

22. Furuno JP, Comer AC, Johnson JK, Rosenberg JH, Moore SL, MacKenzie TD, et al. Using antibiograms to improve antibiotic prescribing in skilled nursing facilities. Infect

Control Hosp Epidemiol [Internet]. 2014 Oct [cited 2022 Oct 5];35 Suppl 3(S3):S56–61. Available from: https://pubmed.ncbi.nlm.nih.gov/25222899/

23. World Health Organization; WHO Methodology for Point Prevalence Survey on Antibiotic Use in Hospitals [Internet]. [cited 2023 Sep 9]. Available from: https://www.who.int/publications/i/item/WHO-EMP-IAU-2018.01

24. Ministry of Health Uganda; Kiruddu Referral Hospital [Internet]. 2022 [cited 2022 Jan 1]. Available from: https://www.kiruddu.hosp.go.ug/

25. Pfizer. Pfizer and Wellcome Launch Surveillance Program to Combat Growing Threat of Antimicrobial Resistance in Sub-Saharan Africa | Pfizer [Internet]. 2020 [cited 2023 Feb 19]. Available from: https://www.pfizer.com/news/press-release/press-release-detail/pfizer-and-wellcome-launch-surveillance-program-combat

26. New Vision Uganda; Germany donates hi-tech laboratory equipment to Kiruddu Hospital - New Vision Official. 2022 [cited 2023 Feb 19]; Available from: https://www.newvision.co.ug/category/news/germany-donates-hi-tech-laboratory-equipment-136815

27. GLOHRA; Strengthening Development Cooperation Partnerships: Global Health [Internet]. 2022 [cited 2023 Sep 10]. Available from: https://globalhealth.de/strengthening-development-cooperation-partnerships.html

28. National Medical Stores; Home - National Medical Stores [Internet]. [cited 2023 Sep 10]. Available from: https://www.nms.go.ug/

29. Browne AJ, Chipeta MG, Haines-Woodhouse G, Kumaran EPA, Hamadani BHK, Zaraa S, et al. Global antibiotic consumption and usage in humans, 2000-18: a spatial modelling study. Lancet Planet Health [Internet]. 2021 Dec 1 [cited 2023 Dec 5];5(12):e893–904. Available from: https://pubmed.ncbi.nlm.nih.gov/34774223/

30. Uganda Bureau of Statistics. Uganda Bureau of Statistics. 2022 [cited 2024 Jan 15]. 2022 Statistical Abstract - Uganda Bureau of Statistics. Available from: https://www.ubos.org/2022-statistical-abstract/

31. World Health Organization; Defined Daily Dose (DDD) [Internet]. [cited 2023 Sep 10]. Available from: https://www.who.int/tools/atc-ddd-toolkit/about-ddd

32. Ministry of Health Uganda; Uganda Clinical Guidelines-National Guidelines for Management of Common Conditions [Internet]. Ministry of Health Kampala; 2016. Available from: https://health.go.ug/sites/default/files/Uganda Clinical Guidelines 2016_FINAL.pdf

33. Murungi M, Ndagije HB, Kiggundu R, Kesi DN, Waswa JP, Rajab K, et al. Antimicrobial consumption surveillance in Uganda: Results from an analysis of national import data for the human health sector, 2018-2021. J Infect Public Health [Internet]. 2023 Dec 1 [cited 2023 Dec 5];16 Suppl 1. Available from: https://pubmed.ncbi.nlm.nih.gov/37926595/

34. World Health Organization; GLASS manual on the management of antimicrobial consumption data. Who [Internet]. 2020 [cited 2023 Dec 5];iv, 72 p. Available from: https://www.who.int/publications/i/item/9789240000421

35. Mthombeni TC, Burger JR, Lubbe MS, Julyan M. Antibiotic consumption in the public sector of the Limpopo province, South Africa, 2014–2018. S Afr J Infect Dis [Internet]. 2022 Oct 25 [cited 2024 Jan 30];37(1):462. Available from: /pmc/articles/PMC9634943/

36. Maina M, Mwaniki P, Odira E, Kiko N, McKnight J, Schultsz C, et al. Antibiotic use in Kenyan public hospitals: Prevalence, appropriateness and link to guideline availability. International Journal of Infectious Diseases [Internet]. 2020 Oct 1 [cited 2024 Jan 30];99:10. Available from: /pmc/articles/PMC7562818/

37. Mbwasi R, Mapunjo S, Wittenauer R, Valimba R, Msovela K, Werth BJ, et al. National Consumption of Antimicrobials in Tanzania: 2017-2019. Front Pharmacol [Internet]. 2020 Oct 30 [cited 2023 Dec 5];11. Available from: https://pubmed.ncbi.nlm.nih.gov/33192526/

38. Namugambe JS, Delamou A, Moses F, Ali E, Hermans V, Takarinda K, et al. National Antimicrobial Consumption: Analysis of Central Warehouses Supplies to In-Patient Care Health Facilities from 2017 to 2019 in Uganda. Trop Med Infect Dis [Internet]. 2021 [cited 2023 Dec 5];6(2). Available from: https://pubmed.ncbi.nlm.nih.gov/34069434/

39. Tirfe M, Alemu A, Alemu W, Woldearegay M, Asfaw G, Gerba H, et al. A three years antimicrobials consumption in Ethiopia from 2017 to 2019: A cross- sectional study. PLoS One [Internet]. 2023 Apr 1 [cited 2023 Dec 5];18(4). Available from: https://pubmed.ncbi.nlm.nih.gov/37023072/

40. World Health Organization; Global action plan on antimicrobial resistance [Internet]. 2016 [cited 2022 Oct 5]. Available from: https://www.who.int/publications/i/item/9789241509763

41. Sharland M, Gandra S, Huttner B, Moja L, Pulcini C, Zeng M, et al. Encouraging AWaRe-ness and discouraging inappropriate antibiotic use-the new 2019 Essential Medicines List becomes a global antibiotic stewardship tool. Lancet Infect Dis [Internet]. 2019 Dec 1 [cited 2024 Feb 27];19(12):1278–80. Available from: https://pubmed.ncbi.nlm.nih.gov/31782385/

42. World Health Organization; The WHO AWaRe (Access, Watch, Reserve) antibiotic book [Internet]. 2022 [cited 2023 Sep 7]. Available from: https://www.who.int/publications/i/item/9789240062382

43. Cantón R, Morosini MI. Emergence and spread of antibiotic resistance following exposure to antibiotics. FEMS Microbiol Rev [Internet]. 2011 Sep [cited 2023 Dec 5];35(5):977–91. Available from: https://pubmed.ncbi.nlm.nih.gov/21722146/

44. Uganda National Academy of Sciences; Antibiotic Resistance in Uganda: Situation Analysis and Recommendations UGANDA NATIONAL ACADEMY OF SCIENCES [Internet]. 2015. Available from: www.ugandanationalacademy.org

45. Kalyesubula Id R, Id TR, Andia-Biraro I, Alupo P, Kimuli I, Nabirye S, et al. Trends of admissions and case fatality rates among medical in-patients at a tertiary hospital in Uganda; A four-year retrospective study. 2019; Available from: https://doi.org/10.1371/journal.pone.0216060

46. Kiguba R, Karamagi C, Bird SM. Extensive antibiotic prescription rate among hospitalized patients in Uganda: but with frequent missed-dose days. J Antimicrob Chemother [Internet]. 2016 Jun 13 [cited 2023 Sep 10];71(6):1697–706. Available from: https://pubmed.ncbi.nlm.nih.gov/26945712/

47. Kemigisha E, Nanjebe D, Ii YB, Langendorf C, Aberrane S, Nyehangane D, et al. Antimicrobial treatment practices among Ugandan children with suspicion of central nervous system infection. PLoS One [Internet]. 2018 Oct 1 [cited 2023 Sep 10];13(10). Available from: https://pubmed.ncbi.nlm.nih.gov/30300411/

48. Anand Paramadhas BD, Tiroyakgosi C, Mpinda-Joseph P, Morokotso M, Matome M, Sinkala F, et al. Point prevalence study of antimicrobial use among hospitals across Botswana; findings and implications. Expert Rev Anti Infect Ther [Internet]. 2019 Jul 3 [cited 2023 Sep 10];17(7):535–46. Available from: https://pubmed.ncbi.nlm.nih.gov/31257952/

49. Van De Sande-Bruinsma N, Grundmann H, Verloo D, Tiemersma E, Monen J, Goossens H, et al. Antimicrobial drug use and resistance in Europe. Emerg Infect Dis [Internet]. 2008 [cited 2023 Sep 10];14(11):1722–30. Available from: https://pubmed.ncbi.nlm.nih.gov/18976555/

50. Nanyunja D, Chothia MY, Opio KC, Ocama P, Bwanga F, Kiggundu D, et al. Incidence, microbiological aspects and associated risk factors of catheter-related bloodstream infections in adults on chronic haemodialysis at a tertiary hospital in Uganda. IJID regions [Internet]. 2022 Dec [cited 2023 Jun 12];5:72–8. Available from: https://pubmed.ncbi.nlm.nih.gov/36212918/

51. Sharland M, Gandra S, Huttner B, Moja L, Pulcini C, Zeng M, et al. Encouraging AWaRe-ness and discouraging inappropriate antibiotic use-the new 2019 Essential Medicines List becomes a global antibiotic stewardship tool. Lancet Infect Dis [Internet]. 2019 Dec 1 [cited 2023 Sep 10];19(12):1278–80. Available from: https://pubmed.ncbi.nlm.nih.gov/31782385/

52. World Health Organization; WHO access, watch, reserve (AWaRe) classification of antibiotics for evaluation and monitoring of use, 2021 [Internet]. 2021 [cited 2023 Sep 10]. Available from: https://apps.who.int/iris/handle/10665/345555

53. Rudd KE, Tutaryebwa LK, West TE. Presentation, management, and outcomes of sepsis in adults and children admitted to a rural Ugandan hospital: A prospective observational cohort study. PLoS One [Internet]. 2017 Feb 1 [cited 2023 Sep 10];12(2). Available from: https://pubmed.ncbi.nlm.nih.gov/28199348/

54. Gutema G, Håkonsen H, Engidawork E, Toverud EL. Multiple challenges of antibiotic use in a large hospital in Ethiopia - a ward-specific study showing high rates of hospital-acquired infections and ineffective prophylaxis. BMC Health Serv Res [Internet]. 2018

May 3 [cited 2023 Sep 10];18(1). Available from: https://pubmed.ncbi.nlm.nih.gov/29724214/

55. Lyamuya F, Muro F, Sheng T, Mallya R, Uronu TS, Minde RM, et al. 2029. Prevalence of Antibiotic Use and Administration among Hospitalized Adult Patients at a Tertiary Care Hospital in Kilimanjaro, Tanzania. Open Forum Infect Dis [Internet]. 2019 Oct 23 [cited 2023 Sep 10];6(Suppl 2):S681. Available from: /pmc/articles/PMC6808873/

56. World Health Organization; WHO Model Lists of Essential Medicines [Internet]. [cited 2023 Sep 10]. Available from: https://www.who.int/groups/expert-committee-on-selection-and-use-of-essential-medicines/essential-medicines-lists

57. Ministry of Health Uganda; MANUAL FOR MANAGEMENT AND CONTROL OF TUBERCULOSIS AND LEPROSY 3RD EDITION MARCH 2017 - Ministry of Health | Government of Uganda [Internet]. [cited 2023 Sep 11]. Available from: https://www.health.go.ug/cause/manual-for-management-and-control-of-tuberculosis-and-leprosy-3rd-edition-march-2017/

58. Atuhaire J, Nakitto D, Kiguba R, Figueras A, Ndagije HB, Serwanga A, et al. Prescription of Levofloxacin and Moxifloxacin in Select Hospitals in Uganda: A Pilot Study to Assess Guideline Concordance. Antibiotics (Basel) [Internet]. 2020 Aug 1 [cited 2023 Sep 10];9(8):1–10. Available from: https://pubmed.ncbi.nlm.nih.gov/32717942/

59. Obakiro SB, Napyo A, Wilberforce MJ, Adongo P, Kiyimba K, Anthierens S, et al. Are antibiotic prescription practices in Eastern Uganda concordant with the national standard treatment guidelines? A cross-sectional retrospective study. J Glob Antimicrob Resist [Internet]. 2022 Jun 1 [cited 2023 Sep 10];29:513–9. Available from: https://pubmed.ncbi.nlm.nih.gov/34890831/

60. Kiggundu R, Wittenauer R, Waswa JP, Nakambale HN, Kitutu FE, Murungi M, et al. Point Prevalence Survey of Antibiotic Use across 13 Hospitals in Uganda. Antibiotics (Basel) [Internet]. 2022 Feb 1 [cited 2023 Sep 10];11(2). Available from: https://pubmed.ncbi.nlm.nih.gov/35203802/

61. Kagoya EK, Royen K Van, Waako P, Royen P Van, Iramiot JS, Obakiro SB, et al. Experiences and views of healthcare professionals on the prescription of antibiotics in Eastern Uganda: A qualitative study. J Glob Antimicrob Resist [Internet]. 2021 Jun 1 [cited 2023 Sep 10];25:66–71. Available from: https://pubmed.ncbi.nlm.nih.gov/33667701/

62. Seni J, Mapunjo SG, Wittenauer R, Valimba R, Stergachis A, Werth BJ, et al. Antimicrobial use across six referral hospitals in Tanzania: a point prevalence survey. BMJ Open [Internet]. 2020 Dec 15 [cited 2023 Sep 10];10(12). Available from: https://pubmed.ncbi.nlm.nih.gov/33323448/

63. Skosana PP, Schellack N, Godman B, Kurdi A, Bennie M, Kruger D, et al. A point prevalence survey of antimicrobial utilisation patterns and quality indices amongst hospitals in South Africa; findings and implications. Expert Rev Anti Infect Ther [Internet]. 2021 [cited 2023 Sep 10];19(10):1353–66. Available from: https://pubmed.ncbi.nlm.nih.gov/33724147/

64. Chetty S, Reddy M, Ramsamy Y, Naidoo A, Essack S. Antimicrobial stewardship in South Africa: a scoping review of the published literature. JAC Antimicrob Resist [Internet]. 2019 Dec 1 [cited 2023 Sep 10];1(3). Available from: https://pubmed.ncbi.nlm.nih.gov/34222934/

65. Frumence G, Mboera LEG, Sindato C, Katale BZ, Kimera S, Metta E, et al. The Governance and Implementation of the National Action Plan on Antimicrobial Resistance in Tanzania: A Qualitative Study. Antibiotics (Basel) [Internet]. 2021 Mar 1 [cited 2023 Sep 10];10(3). Available from: https://pubmed.ncbi.nlm.nih.gov/33803077/
